# In Memoriam: Paul J. Lioy

**DOI:** 10.1289/ehp.1510675

**Published:** 2015-09-01

**Authors:** Timothy J. Buckley, Bernard D. Goldstein, Clifford P. Weisel, Junfeng (Jim) Zhang, John Adgate, Panos G. Georgopoulos

Exposure science has lost one of its founders and intellectual champions. Paul J. Lioy passed away unexpectedly on 8 July 2015 at the age of 68. The world has been left a far better place thanks to Lioy’s vision and creativity, enormous scientific contributions, and the many people that he shaped and influenced through teaching, mentoring, and friendships.

Lioy’s vision and creativity took on many forms but was centered on a field of study that became known as exposure science—a science that he was instrumental in founding and developing. In 1985 he joined the Rutgers faculty and set out to build a graduate program in exposure science, the first of its kind in the world, within the university’s Environmental and Occupational Health Sciences Institute. In 1991 he chaired the National Research Council (NRC) committee that produced *Human Exposure Assessment for Airborne Pollutants: Advances and Opportunities*, which both initially defined the field and largely established it as a credible line of science and research. In his preface to this report, Lioy wrote about the importance of linking “exposure assessment with the practical application of risk reduction and exposure-mitigation strategies.” He was one of a handful of visionaries who in 1990 founded the premier professional society in exposure science, the International Society of Exposure Science. He went on to serve as one of its first presidents in 1993–1994.

Over the years, and due in no small part to Lioy’s commitment, passion, and intellectual drive, his vision for exposure science has taken root and prospered within government, academia, and private industry. For example, the U.S. Environmental Protection Agency (EPA) now has a National Exposure Research Laboratory, and ExxonMobil Biomedical Sciences has a section named Exposure Science. In 2012 Lioy cochaired a second NRC committee, which produced *Exposure Science in the 21st Century: A Vision and a Strategy*. Today nearly 30 federal agencies participate in the Exposure Science in the 21st Century Federal Working Group established in response to this report. We now have a technical journal devoted to exposure science, the *Journal of Exposure Science and Environmental Epidemiology* (*JESEE*), with a strong and rising impact factor. As deputy editor, Lioy was very influential in guiding *JESEE*’s success. He trained dozens of doctoral students who have gone on to positions in academia, government, consulting, and private industry. Due to his work, vision, and commitment over 40 years, his key role in the founding and development of exposure science is clearly a pillar of his legacy.

Another pillar of Lioy’s legacy is the enormous and powerful body of science that he has left us. There are nearly 300 technical articles to his name. His work was remarkable in its real-world relevance and impact. Some of his early research on air pollution demonstrated that ozone at ambient levels affects children’s respiratory health. This was followed by research that prompted the EPA’s move from a 1-hour to an 8-hour ozone standard so as to better protect children exercising outdoors on warm summer days. Lioy conducted seminal work providing evidence of the importance of indoor sources of pollution, the need for personal monitoring to accurately assess air pollution exposures, and the necessity of a total-exposure monitoring approach to account for contaminants found in multiple environmental media.

Furthermore, Lioy’s research resulted in more precise risk and policy calculations revealing heretofore unrecognized exposure pathways such as dermal absorption during bathing and showering to disinfectant by-products found in tap water, and the uptake of sprayed indoor pesticides into children’s toys, with resultant significant exposure. These studies contributed significantly to the scientific basis for regulations of drinking water and registration of pesticides permitted for indoor use. His work and contributions were consistently highly cited and have received prestigious recognition from his peers in professional societies (the Jerome J. Wesolowski Award for lifetime excellence in exposure assessment research and the Air and Waste Management Association’s Frank A. Chambers Award for outstanding achievement in the science of air pollution control), at the Robert Wood Johnson Medical School (e.g., the R. Walter Schlesinger Basic Science Mentoring Award), and in the community (e.g., the Cranford Chamber of Commerce Meritorious Community Service Award).

As a luminary in the field, he was sought by many scientific organizations that benefited from his critical and creative mind. He served on numerous National Academy of Sciences panels, the U.S. EPA’s Science Advisory Board, and the editorial board for a number of technical journals, including *Environmental Health Perspectives*, *Environmental Research*, and *Atmospheric Environment*, to name a few.

No doubt one of Lioy’s most profound and influential contributions to public health was his response to the 9/11 World Trade Center attacks in 2001. Lioy was deeply moved by this tragedy. Much of what we know about the dust that enveloped the community and workers can be attributed to him; he provided a key “exposure” piece to the public health puzzle of the environmental disaster. This work consumed him for much of a decade and was documented in his book *DUST: The Inside Story of Its Role in the September 11th Aftermath*, which was published in 2010. This book also represented his response to perhaps his greatest frustration: our inability to effectively communicate the role and importance of exposure science to decision makers and the general public. Lioy recognized the need for a cogent plan to shape a public health response to disasters, and this became his research focus, direction, and passion. To ensure that the public and first responders would be protected, he again left the confines of academia and worked hard to bridge the gap between science and policy through efforts with law makers and regulators in Washington, DC, and in New Jersey.

In all he did, Paul Lioy was a passionate man: passionate about his family, his science, his friends, his students, and his favorite sports teams. The world and the scientific community has benefited from his passion for understanding the role of exposure science in protecting the environment and public health.

Lioy has given us much through his vision, creativity, intellect, teaching, mentoring, and friendship. We will treasure the rich legacy of his science and those whom he has touched through his teaching, mentoring, and friendship. His voice and presence will be sorely missed, but he will live on and be celebrated in the science he has left us and through the many of us whom he developed and influenced.

We acknowledge that there are many who could have and would have written or contributed to this tribute. For practical reasons, a small group of us gave it our heartfelt best shot.

